# Illustrating Bayesian Indices of Effect Existence and Practical Significance in Anesthesiology Trials

**DOI:** 10.1097/ALN.0000000000005914

**Published:** 2026-02-03

**Authors:** Markus Huber

**Affiliations:** 1 Bern University Hospital, University of Bern, Bern, Switzerland markus.huber@insel.ch

## To the Editor:

The statistical evidence in randomized controlled trials with respect to treatment efficacy was, and still is, predominantly analyzed from a frequentist perspective.^1^ However, limitations of traditional null hypothesis significance testing, *P* values, and confidence intervals are increasingly appreciated, in particular with respect to the need for statistically correct interpretations of these quantitites.^2^ In search for additional interpretations of statistical evidence that could complement traditional (frequentist) trial analyses, Bayesian methods have increasingly gained momentum in medical research.^3^

A Bayesian approach to trial analysis allows the calculation of a number of different quantities, each considering a particular aspect of the trial’s design assumptions (*e.g.*, test hypothesis) and the collected data. We refer to these quantities as “indices” in what follows, as they summarize the trial’s evidence, each in its own way, in a single, interpretable number.^4^ In the context of hypothesis testing, for instance, one Bayesian index compares the relative probability of the null hypothesis being true *versus* the alternative hypothesis being true before and after the trial. A different example is the estimation of the treatment effect and its uncertainty, where another Bayesian index allows for a probability statement regarding the effect being larger (or smaller) than the null value of zero effect. In this research letter, two such Bayesian indices are illustrated in a suite of trials in anesthesiology. Conceptually, the two indices of choice refer to the notions of effect existence and practical significance, which are defined further below. I first provide a Bayesian perspective on a generic trial and then briefly discuss hypothesis testing and effect estimation from a frequentist and Bayesian perspective. I conclude by discussing the added and complementary value of these two Bayesian indices in interpreting trial results, particularly as a means to move beyond the focus on the null value by considering properties of the full posterior probability distribution of the estimated effect size.

Consider a trial investigating the efficacy of a new treatment (effect size: ∆) within a traditional hypothesis testing framework of a null hypothesis of zero effect (H_0_: ∆ = 0 or a different null value, depending on the chosen effect measure) and alternative hypothesis of nonzero effect (H_1_: ∆ ≠ 0). A frequentist approach considers the effect size as a fixed, unknown parameter. In contrast, a Bayesian approach considers the trial’s parameters (*e.g.*, H_0_, H_1_, and ∆) probabilistically with associated prior probability distributions (Pr(H_0_), Pr(H_1_), and Pr(∆)). These prior distributions can be defined in many ways; for example, by means of elicitation of opinions, external evidence of prior studies, or from a range of default priors (*e.g.*, noninformative priors).^5^ Now, Bayes’ theorem allows quantification of the belief regarding the relative probability of these two complementary hypotheses of being true before and after the trial^6^: $\frac{Pr\left(H_{0}\;|\;data\right)}{Pr\left(H_{1}\;|\;data\right)}=\frac{Pr\left(data\;|\;H_{0}\right)}{Pr\left(data\;|\;H_{1}\right)}\frac{Pr\left(H_{0}\right)}{Pr\left(H_{1}\right)}$ (the posterior odds are equal to the likelihood ratio times the prior odds). Similarly, Bayes’ theorem can be applied to update the prior belief regarding the effect size (Pr(Δ), the prior) to a posterior probability distribution: $Pr\left(\Delta \;|\;data\right)=\frac{Pr\left(data\;|\;\Delta \right)}{Pr\left(data\right)}Pr\left(\Delta \right)$ (the posterior). In the following paragraphs, I briefly discuss frequentist and Bayesian statistics related to hypothesis testing and effect estimation in randomized controlled trials.

With respect to frequentist statistics, the *P* value constitutes a statistical summary measure of the compatibility of the observed data und the predictions (or expectations) from a particular statistical model (*e.g.*, the test hypothesis).^2^ The *P* value is traditionally derived by conditioning on the null hypothesis and considers test statistics at least as large as the observed value of the test statistic: *P* = $Pr\left(test\;statistic\;as\;least\;as\;large\;as\;observed\;|\;H_{0}\right)$. Extending the analysis to different effect sizes beyond the null value of interest results in different *P* values: one *P* value for each considered effect size (by means of its test statistic). The traditional 95% CI summarizes effect sizes that yield *P* values larger than 0.05, providing a convenient summary measure of hypothesis tests for many effect sizes.^2^ In terms of interpretation, the range of effects sizes with *P* > 0.05 (that is, effect sizes within the 95% CI) are considered to be more compatible with the observed data than effect sizes outside that range. Note, however, that the 95% CIs do not provide a probabilistic interpretation of the effect size estimate or of the probabilities of a null or nonzero effect—these probabilistic interpretations require a Bayesian approach.^2^ By construction, the 95% CI would contain the true effect size in 95% of the cases in a suite of hypothetical trial repetitions under identical conditions (the so-called coverage probability).

In terms of Bayesian statistics, we focus on indices based on the posterior distribution of the estimated effect size (Pr(Δ | data)). The posterior distribution allows for formal probability statements of our belief regarding both the magnitude and direction regarding the true, underlying treatment effect based on collected data. So-called credible intervals can be constructed as a means to summarize the posterior distribution; for example, the equal-tailed 95% credible interval provides a range of treatment effects containing the true value with 95% probability. The 95% credible interval can be calculated from the 2.5th and 97.5th percentiles of the posterior distribution. The *probability of direction* represents the certainty regarding the direction of a treatment effect, either positive or negative, and can range from 50 to 100%. This index is defined as the proportion of the posterior distribution of the same sign than the median’s sign. In other words, the index is the larger of the probability that the effect is positive and the probability that the effect is negative. It can be demonstrated that there is an approximate 1:1 correspondence with the associated *P* value: *P*_two-sided_ = 2 × (1 − *probability of direction*). Thus, the *probability of direction* can be considered the *P* value’s Bayesian equivalent.^4^ We refer to this index as one of effect existence, as it quantifies the consistency of the effect estimate in a particular direction without referring to other characteristics, such as effect magnitude or its clinical relevance. Another index is based on the concept of the region of practical equivalence (ROPE). The region of practical equivalence denotes a range of treatment values that can be considered to have practically, rather than purely statistically, no effect. *ROPE*_*full*_ refers to the percentage of the effect size’s posterior distribution inside the region of practical equivalence, thus featuring similar notions as traditional equivalence testing. For example, the region of practical equivalence could be chosen equal to a classical (frequentist) equivalence margin for a particular clinical case. Overall, the smaller the *ROPE*_*full*_, the more significant the treatment effect can be considered for practical purposes in the sense that the treatment effect is expected to be beyond values that can be clinically considered to be equal to the null effect (that is, the region of practical equivalence).

We illustrate these two indices (*probability of direction* and *ROPE*_*full*_) using outcome data in 52 multicenter randomized controlled trials in anesthesiology compiled by Seretny *et al.*^7^ We calculated a Bayesian logistic regression model for each study, with the primary outcome as dependent variable and treatment as covariate. For simplicity, we used weakly informative priors. The region of practical equivalence was conventionally defined as the interval from 0.84 to 1.20 on the odds ratio scale.^4^ *P* values are based on Fisher's exact test. All computations were performed with R version 4.4.2 (https://github.com/marbhuber/bayesian_indices_rcts_anesthesiology; accessed November 1, 2024).

**Fig. 1.**
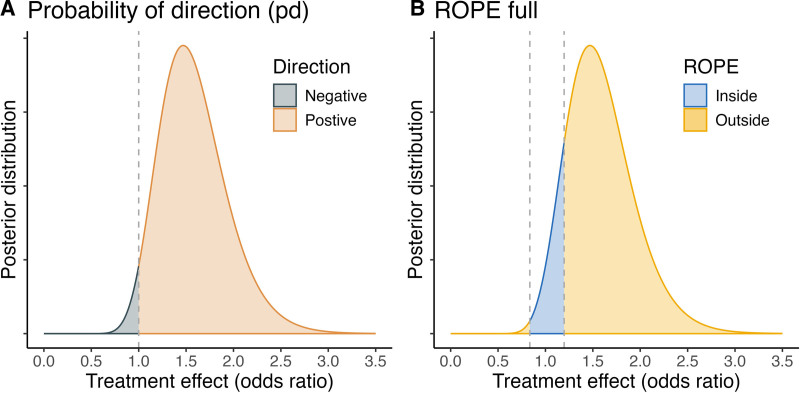
Illustration of the two Bayesian indices *probability of direction* (*A*) and the percentage of the effect size’s posterior distribution inside the region of practical equivalence (*ROPE*_*full*_) (*B*) for a study investigating the efficacy of intravenous amisulpride in the prevention of postoperative nausea and vomiting in adult surgical patients.^8^ *A* and *B* illustrate the posterior distribution of the treatment effect (on the odds ratio scale) derived from a Bayesian logistic regression model with a weakly informative prior. In *A*, the *probability of direction* denotes the proportion of the treatment effect’s posterior distribution that is of the posterior’s median’s sign (*orange*). In *B*, the posterior distribution of the effect estimate is *shaded* according to the region of practical equivalence (ROPE) around the null effect. A default ROPE is chosen, including odds ratios from 0.84 to 1.20.^4^ *ROPE*_*full*_ is defined as the percentage of the *blue-shaded region* of the full posterior distribution.

Figure 1 illustrates the two Bayesian indices for a study investigating the efficacy of intravenous amisulpride in the prevention of postoperative nausea and vomiting in adult surgical patients.^8^ Of 318 patients, 89 of 155 (57.4%) and 76 of 163 (46.6%) experienced a positive outcome in the intervention and placebo group, respectively. By traditional interpretation, superiority could not be demonstrated (odds ratio, 1.54; 95% CI, 0.97 to 2.46; *P* = 0.057). In contrast, the Bayesian analysis results in an equal-tailed 95% credible interval of 0.99 to 2.41 and demonstrates strong evidence of the existence of a treatment effect (*probability of direction*: 97.3%) and its practical significance (*ROPE*_*full*_: 12.7%). This example highlights the added value when shifting the emphasis from hypothesis testing toward estimation.

**Fig. 2.**
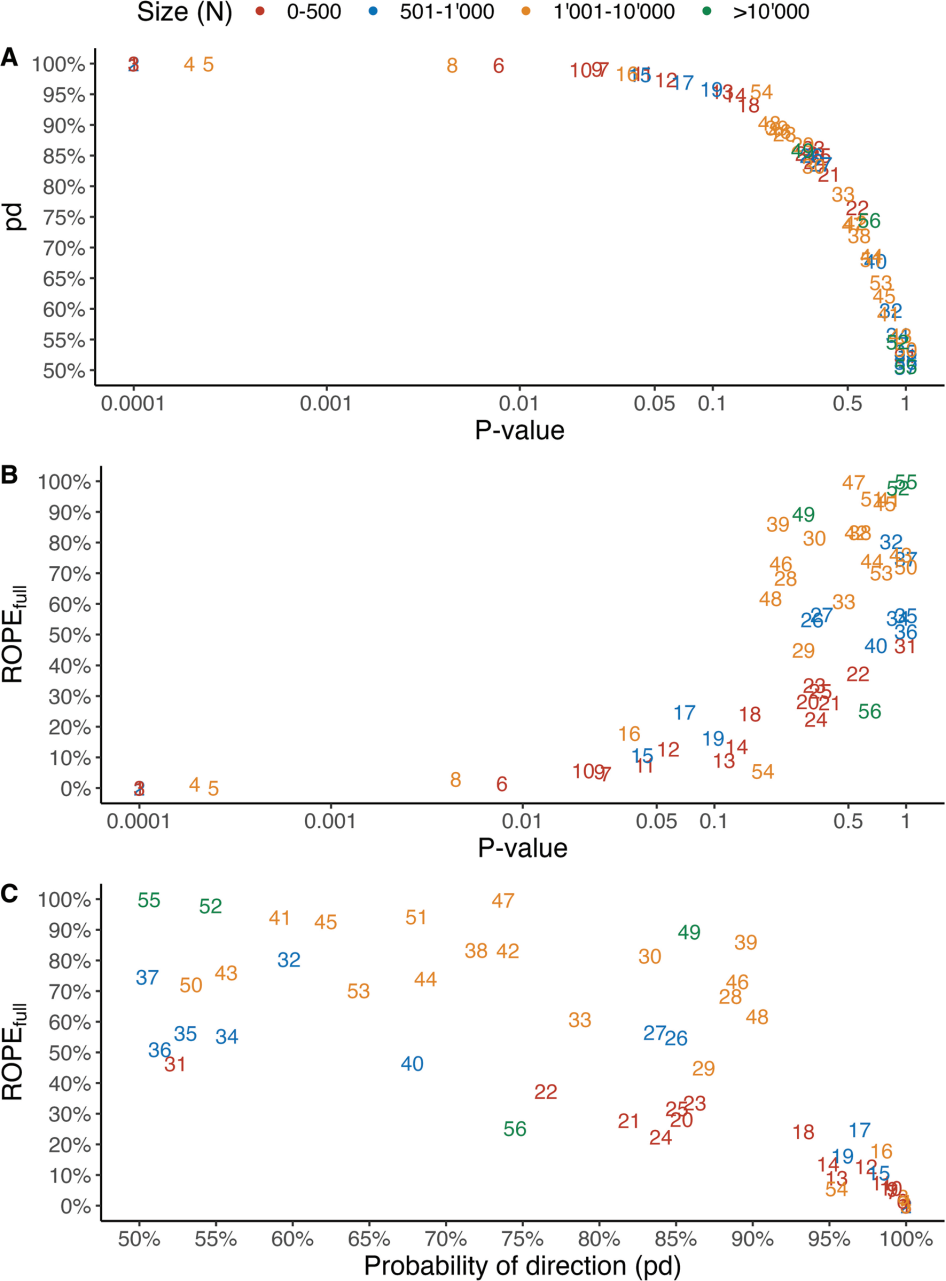
Illustration of the associations of (*A*) the *probability of direction* (pd) index with the *P* value, (*B*) the percentage of the effect size’s posterior distribution inside the region of practical equivalence (*ROPE*_*full*_) index with the *P* value, and (*C*) the pd index and the *ROPE*_*full*_ index in a suite of 52 anesthesiology trials. Note that the *P* values are shown on the log scale for illustration purposes. Each number corresponds to a particular trial, and the study identifiers are listed in the supplemental material of the primary publication by Seretny *et al.*^7^ Note that there are some trials featuring factorial designs or coprimary outcomes, resulting in a total of 56 treatment effect estimates. *Colors* denote the sample size of a study.

The two Bayesian indices for the entire set of studies as well as their associations with corresponding *P* values are shown in figure 2. The median *P* value is 0.33 (interquartile range: 0.07 to 0.67). The corresponding numbers for the *probability of direction* and *ROPE*_*full*_ are 85% (interquartile range: 68 to 97%) and 46% (interquartile range: 12 to 74%), respectively. The approximate 1:1 association of the *probability of direction* index with the *P* value is shown in figure 2A, whereas the *ROPE*_*full*_ index is only weakly associated with the *P* value (fig. 2B). The two indices are weakly associated (fig. 2C); although the probability of effect existence (*probability of direction*) is associated with practical significance (*ROPE*_*full*_), the two indices provide complementary information regarding the evidence of the treatment effect. For high evidence with respect to effect existence and practical significance, the two indices are highly correlated. Further, figure 2C highlights that one can be very confident about the direction of a treatment effect without being confident of whether its magnitude is practically relevant and vice versa. Importantly, figure 2 highlights that the traditional significance level of 0.05 would distinguish between studies featuring very similar evidence regarding the treatment effect. For example, studies 12 (*P* = 0.057 from the example above) and 15 (*P* = 0.042) demonstrate strong and similar evidence of a treatment effect. However, only study 15 would be considered statistically significant.

This short analysis complements a previous analysis of this set of randomized controlled trials by considering the *probability of direction* and the percentage of posterior distribution within the region of practical equivalence (*ROPE*_*full*_).^7^ The previous analysis combined pretest (prior) probabilities of the null and alternative hypothesis (choosing $\frac{Pr\left(H_{0}\right)}{Pr\left(H_{1}\right)}$ equal to 1) with Bayes factors derived from trial data ($\frac{Pr\left(data\;|\;H_{0}\right)}{Pr\left(data\;|\;H_{1}\right)}$) to compute posttest probabilities ($\frac{Pr\left(H_{0}\;|\;data\right)}{Pr\left(H_{1}\;|\;data\right)}$).^7^ The focus of this study is the posterior distribution of the estimated treatment effect, which allows for straightforward statements regarding the probability of different effect sizes, notably about effects beyond the null value by considering the region of practical equivalence. A key finding of this analysis is the demonstration of the complementary value of these indices when interpreting the statistical evidence from a study (fig. 1). In addition, figure 2 highlights that dichotomizing study results into “statistically significant” *versus* “statistically nonsignificant” artificially categorizes studies with very similar evidence regarding the treatment effect. Thus, for the reporting of a single trial, our findings provide a further case for reporting Bayesian indices alongside frequentist measures of evidence.^9^

The study has some limitations. First, we used a default, noninformative prior and the default traditional region of practical equivalence . These limitations could be addressed in further analyses. Second, a detailed discussion of the strength and limitations of each index, notably with respect to sample size, effect presence, and sensitivity to modeling assumptions (*i.e.*, choice of a prior distribution) was beyond the scope of this research letter. However, it is emphasized here that the underlying assumptions and modeling choices should be carefully examined for each analysis, as the values of the indices could be potentially misleading in specific cases. For example, using uninformative or only weakly informative priors in combination with small sample sizes and small effect sizes could potentially overestimate the practical significance of the effect by the *ROPE*_*full*_ index. The reader is referred to the literature for further sensitivity aspects.^4,10^

To conclude, this study illustrates that Bayesian methods offer clinically relevant insights into the analysis of randomized controlled trials. Bayesian indices of effect existence and practical significance provide evidence complementary to traditional *P* values paired with an intuitive interpretation regarding the statistical existence of a treatment effect. Importantly, they allow us to move beyond the statistical significance of *P* values and broaden the focus on treatment effects beyond the null value.

### Acknowledgments

The author thanks Prof. Frank Stüber and Prof. Dominik Günsch (Department of Anaesthesiology and Pain Medicine, Inselspital, Bern University Hospital, University of Bern, Bern, Switzerland) for research support.

### Research Support

Support was provided solely from institutional and/or departmental sources.

### Competing Interests

The author declares no competing interests.
